# Supplementary material for: Prevalence, intensity and associated risk factors of Schistosoma mansoni infections among schoolchildren around Lake Tana, northwestern Ethiopia
Source: PLoS Negl Trop Dis. 2021 Oct 27;15(10):e0009861. doi: 10.1371/journal.pntd.0009861 (PMC8550401; doi:10.1371/journal.pntd.0009861)
Supplement: Attachment — Submitted filename: Reviewreport.docx [file pntd.0009861.s001.docx]

**Prevalence and intensity of *Schistosoma mansoni* infections** 1 **and associate**  **risk factors among schoolchildren around Lake Tana, northwestern Ethiopia**

Comment

The work is important to assess the current status of Schistosomiasis and evaluate the outcome of the MDA intervention in the study area. However, the following points should be considered to improve the manuscript.

1. The justification given to conduct the research, line number 105-108, is not sounding. Hence, this needs correction.
2. In the abstract part, method section, line 28-30, reads as “A questionnaire was used to data on collect socio-demographic information and potential risk factors of *S. mansoni* infection.”. This needs rewriting.
1. In the abstract part, method section, data analysis is done using SPSS version 23 while in the main Method section it is reported that data was analyzed using SPSS version 21 and STATA. This should be consistent.
2. In the abstract part, the conclusion section, line number 47-49, states that “Therefore, integrated control strategies are needed to improve the health conditions as well as the academic performance of schoolchildren in the study area.” A conclusion and recommendation should stem from the research finding. Here, there is no work done on the evaluation of school children academic or school performance. Therefore, this needs amendment.
3. In the background section, there should be explanation about MDA activities practiced in the study areas since MDA was discussed in the discussion section.
4. Sampling and sample size determination: line number 168-169 shows the number of children allocated for each schools. On the basis of quota the number of student assigned for Ura was 25. Putting percentage results for this small number sample will lead to poor conclusion. This needs explanation on the interpretation of the result.
5. On the discussion section line 362 states about open-field defecation. However, in the sociodemographic data collection section of this manuscript, there is no variable about it. Hence, should not be mentioned as a risk factor for this study, though it is a known fact.
