# Supplementary material for: Prevalence, intensity and associated risk factors of Schistosoma mansoni infections among schoolchildren around Lake Tana, northwestern Ethiopia
Source: PLoS Negl Trop Dis. 2021 Oct 27;15(10):e0009861. doi: 10.1371/journal.pntd.0009861 (PMC8550401; doi:10.1371/journal.pntd.0009861)
Supplement: Attachment — Submitted filename: Reviewer report.docx [file pntd.0009861.s002.docx]

**Manuscript: Prevalence and intensity of *Schistosoma mansoni* infections and associated risk factors among schoolchildren around Lake Tana, northwestern Ethiopia**

Tamirat Hailegebriel, Endalkachew Nibret, Abaineh Munshea

**Reviewer’s Report**

- The manuscript is worth to publish if the authors address the issues mentioned below.

1. **Title: Recommended if modified as** “Prevalence, intensity, and associated risk factors of *Schistosoma mansoni* infections among schoolchildren around Lake Tana, northwestern Ethiopia”
2. **Affiliations:**

- ^1^Department of Biology, College of Science, Bahir Dar University, Bahir Dar, Ethiopia
- ^2^Biotechnology Research Institute (BRI), Bahir Dar University, Bahir Dar, Ethiopia

3. **Abstract**

- **Lines 21-22:** Lacks clarity- recommended as “Schistosomiasis is one of the widely distributed neglected tropical diseases. It is a serious public health problem in sub-Saharan Africa.”
- **Line 29**: “questionnaire was used to data on collect socio-demographic information and potential risk”- needs modification as “questionnaire was used to collect data on socio-demographic information and potential risk”
- **Lines 32-33:** “Ritche’s concentration techniques”- needs modification as “Ritchie’s concentration techniques”
- **Lines 38-43:** It is long. Can be reduced as “Among the different determinant factors being male (AOR = ; 95%CI = ; P-value = ), bathing habits (AOR = ; 95%CI = ; P-value = ), and schools in which students attended (AOR = ; 95%CI = ; P-value = ) were significantly associated with S. mansoni infection.
- **General**: Grammar check is recommended.

4. **Background**

- **Line 85:** Schistosomiasis is one of the neglected tropical diseases (NTD) that infects about 236 million. The decimal is above 236.5. Thus, recommended to be modified as close to 237 million.
- The background is not detail. At least should show review the prevalence and associated risk factors of Schistosomiasis in Ethiopia in general, and in northwest Ethiopia in particular.
- Grammar check is recommended

5. **Materials and methods**

- **Study area:** Conditions suitable for the reproduction and multiplication of the snail hosts should be addressed.
- **Study design:** lacks clarity for randomness.
- **Sample size determination:** not clear why you used 30% (P). The authors need to cite their reference they used for this.
- **Line 181-182: needs modification:** The infection intensity was classified as light (1- 99 EPG), moderate (100-399 EPG) and heavy (greater than 400 EPG) according to WHO cut-off values.
- **Quality control:** What about the quality of questionnaire data?
- **Data analysis:** Line 194: The authors mentioned that they used SPSS version 21. But in the abstract they said version 23. Which one is correct? Stata is not mentioned in the abstract.
- **Ethical consideration and consent to participant**
- **Lines 204-205**: Students were participated in the study only after receiving written consent from their family/guardians for students below 18 years of age.
- It is better if the authors include a sub-sub-title “Operational definitions” and define the common terms they used in the manuscript.
- Grammar check is very recommended b/c there are many subject verb disagreements

6. **Results**

- **Table 1:** It is better to put * at p-values < 0.05 and statistical differences be mentioned. For example infection among males is significantly higher than that of females.
- **Table 2:** Similar comment as that of Table 1.
- **Sub-**title is required above Table 3.
- **Table 3** needs more clarification
- It is better to replace sex of students with gender of students throughout the manuscript
- Table 4 includes the COR. So what is the importance of the X^2^_ analysis? It is recommended to avoid results of the X^2^_ analysis.
- **Lines 277-278:** Factors with a *p*-value < 0.25 were subjected to multivariate logistic regression analysis in order to control for confounding effects.
- **Lines 283-**285: School wise, students attending Qunzela primary school were 10.5 times more likely to be infected with *S. mansoni* compared to students from the other schools (AOR=10.545, 95% CI: 3.264 -34.067, *p* = 0.001)
- **General:** Grammar checks are recommended

7. **Discussion**

- **Line 300:** The present study revealed that 34.9% (95% CI: 31.4-38.7%). The authors should compare and contrast this finding with the works of other researchers.
- Generally, the discussion part needs more comparisons and justifications.
- **NB**. Major modifications are required in this part.

8. **Conclusion**

- Seems repetition of the results section.

9. **References**

- Some inconsistencies e.g., Some journal names are abbreviated and others not
