# Supplementary material for: Prevalence, intensity and associated risk factors of Schistosoma mansoni infections among schoolchildren around Lake Tana, northwestern Ethiopia
Source: PLoS Negl Trop Dis. 2021 Oct 27;15(10):e0009861. doi: 10.1371/journal.pntd.0009861 (PMC8550401; doi:10.1371/journal.pntd.0009861)
Supplement: Attachment — Submitted filename: Response to reviewer comments.docx [file pntd.0009861.s003.docx]

**Response to editor and reviewers comments**

**Dear Editors,**

We are very grateful and appreciate the willingness to review our manuscript and forward constructive comments and suggestions to our manuscript by the reviewers. The comments and suggestions are very vital for the improvement of the manuscript. We have taken all these into consideration and revised the manuscript by incorporating all comments and suggestions. All the changes in the main document are indicated with track change. We have carefully addressed all specific comments and suggestions point by point and indicated with blue colour.

**Editor comments,**

**Comment 1:** Please ensure that the CRediT author contributions listed for every co-author are completed accurately and in full.

**Response:** The contributions of all authors are included in the manuscript (author contribution section). The addition of Zena Ameha is agreed and confirmed by all authors. **Comment 2:** Please provide separate figure files in .tif or .eps format.

Response: The figure is converted to tif format and uploads in the submission system.

**Comment 3:** Please ensure that all author names are spelled correctly in the manuscript file

**Response:** Thank you for the comment. The names of all authors are spelled correctly in the manuscript as well as in the submission form.

**Comment 4:** We noticed that you used "unpublished data" in the manuscript.

**Response:** Thank you for the comment. Information related to unpublished reference is removed from the paper.

**Comment 5:** We have noticed that you have uploaded supporting information but you have not included a list of legends.

**Response:** Legend is included at the end of the references (the legend consists of supporting information 1 and Figure 1).

**Reviewer 1**

The work is important to assess the current status of Schistosomiasis and evaluate the outcome of the MDA intervention in the study area. However, the following points should be considered to improve the manuscript.

1. The justification given to conduct the research, line number 105-108, is not sounding. Hence, this needs correction.

**Response:** The justifications are properly modified in the revised manuscript.

2. In the abstract part, method section, line 28-30, reads as “A questionnaire was used to data on collect socio-demographic information and potential risk factors of *S. mansoni* infection.”. This needs rewriting.

**Response:** the comment is right. A correction has been made in the revised manuscript.

3. In the abstract part, method section, data analysis is done using SPSS version 23 while in the main Method section it is reported that data was analyzed using SPSS version 21 and STATA. This should be consistent.

**Response:** the comment is correct. The data analysis was conducted using SPSS version 23. SPSS version 21 and Stata are removed from the method section in the revised manuscript.

4. In the abstract part, the conclusion section, line number 47-49, states that “Therefore, integrated control strategies are needed to improve the health conditions as well as the academic performance of schoolchildren in the study area.” A conclusion and recommendation should stem from the research finding. Here, there is no work done on the evaluation of school children academic or school performance. Therefore, this needs amendment.

**Response:** Yes the comment is right. Part of the recommendation is removed and modified in the revised manuscript.

5. In the background section, there should be explanation about MDA activities practiced in the study areas since MDA was discussed in the discussion section.

**Response:** Background information about MDA in Ethiopia and the study areas is incorporated in the revised manuscript.

6. Sampling and sample size determination: line number 168-169 shows the number of children allocated for each schools. On the basis of quota the number of student assigned for Ura was 25. Putting percentage results for this small number sample will lead to poor conclusion. This needs explanation on the interpretation of the result.

**Response:** The total population was divided by the sample size to obtain the proportion (7248/710=0.098). The number of students allocated to each school was solely based on proportion to the number of students attending each school. The number of students attending Ura primary school was 255. Therefore, 0.098x255= 25 students were selected from Ura primary school based on the proportion.

7. On the discussion section line 362 states about open-field defecation. However, in the sociodemographic data collection section of this manuscript, there is no variable about it. Hence, should not be mentioned as a risk factor for this study, though it is a known fact.

**Response:** It is a known fact that open-field defecation is directly linked with high schistosome infection. We did not use open-field defecation as a risk factor for schistosome infection in this study. We used open-field defecation as a justification for the possible source for open water infestation with *S. mansoni* cercaria.

**Reviewer 2**

- The manuscript is worth to publish if the authors address the issues mentioned below.

1. **Title: Recommended if modified as** “Prevalence, intensity, and associated risk factors of *Schistosoma mansoni* infections among schoolchildren around Lake Tana, northwestern Ethiopia”

**Response:** Thank you for the recommendation. We have modified the title based on the comment.

2. **Affiliations:**

- ^1^Department of Biology, College of Science, Bahir Dar University, Bahir Dar, Ethiopia
- **Response:** Thanks for the comment; a correction has been made in the revised manuscript.
- ^2^Biotechnology Research Institute (BRI), Bahir Dar University, Bahir Dar, Ethiopia
- **Response:** Thanks for the comment; a correction has been made in the revised manuscript.

3. **Abstract**

- **Comment: Lines 21-22:** Lacks clarity- recommended as “Schistosomiasis is one of the widely distributed neglected tropical diseases. It is a serious public health problem in sub-Saharan Africa.”
- **Response**: A correction has been made in the revised manuscript.
- **Comment: Line 29**: “questionnaire was used to data on collect socio-demographic information and potential risk”- needs modification as “questionnaire was used to collect data on socio-demographic information and potential risk”
- **Response:** Sorry for the mistake. A correction has been made in the revised manuscript.
- **Comment: Lines 32-33:** “Ritche’s concentration techniques”- needs modification as “Ritchie’s concentration techniques”
- **Response:** thank you for the comment, it has been made in the revised manuscript..
- **Comment: Lines 38-43:** It is long. Can be reduced as “Among the different determinant factors being male (AOR = ; 95%CI = ; P-value = ), bathing habits (AOR = ; 95%CI = ; P-value = ), and schools in which students attended (AOR = ; 95%CI = ; P-value = ) were significantly associated with S. mansoni infection.
- **Response:** The results are modified based on the suggestions.
- **General**: Grammar check is recommended.
- **Response**: the grammar is properly checked.

4. **Background**

- **Line 85:** Schistosomiasis is one of the neglected tropical diseases (NTD) that infects about 236 million. The decimal is above 236.5. Thus, recommended to be modified as close to 237 million.
- **Response:** Yes, it is corrected.
- The background is not detail. At least should show review the prevalence and associated risk factors of Schistosomiasis in Ethiopia in general, and in northwest Ethiopia in particular.
- **Response:** The background information is properly updated in the revised paper.
- Grammar check is recommended

**Response:** the grammar is properly checked.

5. **Materials and methods**

- **Study area:** Conditions suitable for the reproduction and multiplication of the snail hosts should be addressed.
- **Response:** A correction has been made in the revised manuscript.
- **Study design:** lacks clarity for randomness
- **Response:** Individual target students were selected by systematic random sampling using intervals obtained by dividing the total source population by the sample size (7248/710=10.2). The first number was obtained by lottery method from 1-10 and the remaining students were selected at every 10 student interval using the class roster until by the last interval of the last section.
- **Sample size determination:** not clear why you used 30% (P). The authors need to cite their reference they used for this.
- **Response:** 30% indicates the previous report on *S. mansoni* prevalence in few schools around Lake Tana. **Reference number 15** in the paper indicates the previous study, which is used for sample size calculation.
- **Line 181-182: needs modification:** The infection intensity was classified as light (1- 99 EPG), moderate (100-399 EPG) and heavy (greater than 400 EPG) according to WHO cut-off values.
- **Response:** The sentence is modified per the comment.
- **Comment: Quality control:** What about the quality of questionnaire data?
- **Response:** The questionnaire was pretested in 5% of students attending nearby schools with similar socio-demographic and environmental conditions but not included in this study. It is included in the revised manuscript.
- **Comment: Data analysis:** Line 194: The authors mentioned that they used SPSS version 21. But in the abstract they said version 23. Which one is correct? Stata is not mentioned in the abstract.
- **Response:** Sorry for the inconsistent in writing. SPSS version 23 was used to analyse the data. SPSS version 21 and Stata has been removed from the revised paper.
- **Comment: Ethical consideration and consent to participant**
- Response: A correction has been made in the revised manuscript.
- **Comment: Lines 204-205**: Students were participated in the study only after receiving written consent from their family/guardians for students below 18 years of age.
- **Response**: Yes, 6 students were above 18 years of age. We are described schoolchildren throughout the manuscript we did not mention school-aged children. Delay in initial starting of the formal education or dropout schooling due to various family factors, students may not complete school at 18 years of age in Ethiopia.
- **Comment:** It is better if the authors include a sub-sub-title “Operational definitions” and define the common terms they used in the manuscript.
- **Response:** Common terms are included in the revised manuscript.
- **Comment:** Grammar check is very recommended b/c there are many subject verb disagreements
- **Response:** We try our best to improve the grammar.

6. **Results**

- **Comment: Table 1:** It is better to put * at p-values < 0.05 and statistical differences be mentioned. For example infection among males is significantly higher than that of females.
- **Response:** A correction has been made in the revised manuscript.
- **Comment: Table 2:** Similar comment as that of Table 1.
- **Response:** A correction has been made in the revised manuscript.
- **Comment: Sub-**title is required above Table 3.
- **Response:** sub-title is incorporated based on the comments.
- **Comment: Table 3** needs more clarification
- **Response:** Table 3 deals with the parasitological findings obtained using Kato-Katz faecal smear and Ritchie concentration techniques among schoolchildren in the study area. We include some information to make it clear for the readers.
- **Comment:** It is better to replace sex of students with gender of students throughout the manuscript
- **Response:** A correction has been made in the revised manuscript.
- **Comment:** Table 4 includes the COR. So what is the importance of the X^2^_ analysis? It is recommended to avoid results of the X^2^_ analysis.
- **Response:** the X^2^- analysis was removed from Tables 1 and 2.
- **Comment: Lines 277-278:** Factors with a *p*-value < 0.25 were subjected to multivariate logistic regression analysis in order to control for confounding effects.
- **Response:** A correction has been made in the revised manuscript.
- **Comment: Lines 283-**285: School wise, students attending Qunzela primary school were 10.5 times more likely to be infected with *S. mansoni* compared to students from the other schools (AOR=10.545, 95% CI: 3.264 -34.067, *p* = 0.001)
- **Response:** The reference was Ura primary school (it is not urban). This school is located in Zegie Peninsula and lower *S. mansoni* prevalence as compared with the remaining schools. We used Ura primary school as a reference.
- **Comment:** Grammar checks are recommended
- **Response:** the grammar is properly checked.

7. **Discussion**

- **Comment: Line 300:** The present study revealed that 34.9% (95% CI: 31.4-38.7%). The authors should compare and contrast this finding with the works of other researchers.

**Response:** The result is properly discussed in the revised manuscript.

- **Comment:** Generally, the discussion part needs more comparisons and justifications. **NB**. Major modifications are required in this part.
- **Response:** the discussion part is significantly improved in the revised manuscript.

8. **Conclusion**

- **Comment:** Seems repetition of the results section.
- **Response:** The conclusion part is modified based on the comment.

9. **References**

- Some inconsistencies e.g., Some journal names are abbreviated and others not
- **Response:** the journal names are properly updated by abbreviated all journal names.
